# Is the beck anxiety inventory a good tool to assess the severity of anxiety? A primary care study in The Netherlands study of depression and anxiety (NESDA)

**DOI:** 10.1186/1471-2296-12-66

**Published:** 2011-07-04

**Authors:** Anna DT Muntingh, Christina M van der Feltz-Cornelis, Harm WJ van Marwijk, Philip Spinhoven, Brenda WJH Penninx, Anton JLM van Balkom

**Affiliations:** 1 Netherlands Institute of Mental Health and Addiction (Trimbos Institute), PO Box 725, Utrecht, 3500 AS, The Netherlands; 2 EMGO Institute for Health and Care Research (EMGO+), PO Box 7057, Amsterdam, 1007 MB, The Netherlands; 3 Department of General Practice, VU University Medical Centre, Van der Boechorststraat 7, Amsterdam, 1081 BT, The Netherlands; 4 Department of Developmental, Clinical and Cross-cultural Psychology, Tilburg University, PO Box 90153, Tilburg, 5000 LE, The Netherlands; 5 Academic Psychiatry Department GGZ Breburg, Lage Witsiebaan 4, Tilburg, 5042 DA, The Netherlands; 6 Institute of Psychology, Leiden University, PO Box 9555 Leiden, 2300 RB, The Netherlands; 7 Department of Psychiatry, Leiden University Medical Centre, PO Box 9600, Leiden, 2300 RC, The Netherlands; 8 Department of Psychiatry, VU University Medical Centre, A.J. Ernststraat 1187 Amsterdam, 1081 HL, The Netherlands; 9 Department of Psychiatry, University Medical Centre Groningen, PO Box 30.001 Groningen, 9700 RB, The Netherlands

## Abstract

**Background:**

Appropriate management of anxiety disorders in primary care requires clinical assessment and monitoring of the severity of the anxiety. This study focuses on the Beck Anxiety Inventory (BAI) as a severity indicator for anxiety in primary care patients with different anxiety disorders (social phobia, panic disorder with or without agoraphobia, agoraphobia or generalized anxiety disorder), depressive disorders or no disorder (controls).

**Methods:**

Participants were 1601 primary care patients participating in the Netherlands Study of Depression and Anxiety (NESDA). Regression analyses were used to compare the mean BAI scores of the different diagnostic groups and to correct for age and gender.

**Results:**

Patients with any anxiety disorder had a significantly higher mean score than the controls. A significantly higher score was found for patients with panic disorder and agoraphobia compared to patients with agoraphobia only or social phobia only. BAI scores in patients with an anxiety disorder with a co-morbid anxiety disorder and in patients with an anxiety disorder with a co-morbid depressive disorder were significantly higher than BAI scores in patients with an anxiety disorder alone or patients with a depressive disorder alone. Depressed and anxious patients did not differ significantly in their mean scores.

**Conclusions:**

The results suggest that the BAI may be used as a severity indicator of anxiety in primary care patients with different anxiety disorders. However, because the instrument seems to reflect the severity of depression as well, it is not a suitable instrument to discriminate between anxiety and depression in a primary care population.

## Background

In primary care, many patients present with anxiety symptoms but these are seldom systematically assessed [1]. To improve anxiety management, assessment of the severity of the anxiety (and subsequent monitoring) is recommended by researchers and also in clinical guidelines [2-4]. With regard to depression, the use of severity indicators in primary care is supported by the results of studies showing that patients value the use of questionnaires as a supplement to the diagnosis made by their general practitioner and as evidence that their problems are taken seriously [5]. Furthermore, when questionnaires to assess severity are used, higher severity scores are related to better care (i.e. higher prescription rates of antidepressant medication and increased referral to secondary care) [6]. Moreover, in some countries incentives are offered when a validated instrument is used at the start of and during the treatment of patients diagnosed with depression [7]. For similar reasons the use of severity scales to assess anxiety symptoms in primary care might be advocated. However, we first have to determine which questionnaires can be used as severity indicators in primary care and what their characteristics are.

As anxiety disorders differ in type and symptoms, assessing the severity of anxiety in general may be more difficult than assessing the severity of depression. General rating scales may not be specific enough to assess the severity of a specific anxiety disorder (i.e. panic disorder or generalized anxiety disorder). However, extensive testing for different forms of anxiety is also not feasible during the short consultations in primary care. Considering its brevity, simplicity, and presumed ability to measure general anxiety, the Beck Anxiety Inventory (BAI) [8] might be a good candidate for use as a severity indicator. Since its development, the BAI has been widely used in clinical research in mental health care, mainly as a measure of general anxiety [9].

However, the BAI has been disputed for its focus on psychophysiological symptoms linked to panic. The results of several studies have found that patients with panic disorder score higher on the BAI than patients with for example generalized anxiety disorder [10-13]. Either way, patients with panic disorder and patients with other anxiety disorders have been found to score significantly higher than patients with no anxiety disorder [14-16]. Remarkably, no study has specifically investigated the co-morbidity of anxiety disorders and how this influences BAI scores, even though co-morbidity occurs frequently [17]. Furthermore, none of the previous BAI studies have focused on primary care populations.

Another presumed quality of the BAI is its ability to discriminate anxiety from depression [8]. Even though in primary care this might be of less importance than in research settings, it is important to know whether the BAI only measures anxiety or whether it is also sensitive to depressive symptomatology. The results of earlier studies suggest a substantial overlap of the BAI with depressive symptoms, illustrated by a moderate correlation between the BAI and depression scales [18]. In terms of differences in the BAI scores of anxious and depressed patients, a large difference was found in the original validation study [8], but in two later studies no difference was found. However, in these studies the authors questioned the results because of limitations in the methodology [15,19].

In the present study, we investigated whether the BAI reflects the severity of anxiety in primary care patients with different anxiety disorders. The mean scores of several patient groups were compared: healthy controls, patients with one anxiety disorder, patients with multiple anxiety disorders, patients with one depressive disorder, and patients with co-morbid anxiety-depression. The diagnostic groups were separated into patients with no co-morbidity and patients with co-morbidity, to ensure homogeneity of the groups. It was hypothesized that the BAI scores of patients with an anxiety disorder would be higher than the BAI scores of healthy controls or depressed patients. Patients with a panic disorder were expected to score higher than patients in the other anxiety disorder groups. We also expected patients with co-morbid disorders to score higher than patients with no co-morbidity.

## Methods

### Participants

The participants in this study were recruited for a large cohort study: the Netherlands Study of Depression and Anxiety (NESDA) [20]. From the baseline sub-sample of 1601 primary care patients in the NESDA cohort we selected all patients with a current anxiety or depressive disorder according to the WHO Composite Interview Diagnostic Instrument (CIDI lifetime version 2.1) and patients with no history of anxiety or depression. DSM-IV classifications of diagnoses within the past month were used to assure present symptomatology. Patients with a history of anxiety or depression, but no current diagnosis, were excluded from the analysis. The mean BAI scores of patients with an anxiety disorder (*N *= 276) and patients with a depressive disorder (*N *= 155), were compared to the mean BAI scores of a control group of patients with no history of anxiety or depressive disorders (*N*= 513). The NESDA study protocol was approved by the Medical Ethics Committee of the VU University Medical Centre.

### Procedures

The primary care sample in the NESDA study was recruited between September 2004 and February 2007 through 65 general practitioners situated in different parts of the Netherlands (Amsterdam, Groningen, and Leiden). A screening questionnaire was sent to 23750 patients between 18 and 65 years of age who had consulted their general practitioner in the past four months. This questionnaire consisted of the Kessler-10 (K-10) [21], which screens for affective disorders, supplemented with five questions about anxiety (Extended K-10, or EK-10). The EK-10 showed adequate psychometric properties, with a sensitivity of .90 and a specificity of .75 to detect anxiety or depressive disorders [22]. Participants who returned the EK-10 (*N *= 10706, 45.9%), scored positively (*N *= 4592, 43%), gave informed consent (*N *= 3420, 74%) and could be contacted (*N *= 2995, 88%) had a telephone screening interview based on short-form sections of the CIDI (major depression, dysthymia, social phobia, panic disorder, agoraphobia, and generalized anxiety disorder).

Patients who were unwilling to be interviewed (*N *= 267, 9%), were not fluent in Dutch (*N *= 86, 3%) or were being treated in a mental health organization (*N *= 155, 5%), were excluded. All other patients who screened positive on the telephone screening (*N *= 1162, 47%) and a random sample of patients who screened negative (*N *= 924) were contacted for a face-to-face interview. As 437 (24%) participants were unwilling to participate and 39 (2%) could not be contacted or were not fluent in Dutch, 1610 primary care patients were finally included in the NESDA study and completed the baseline assessment. More details about the recruitment process are described elsewhere [20]. Of the 1610 NESDA participants, 9 patients who did not complete the BAI were excluded from the analysis. The present sample therefore consisted of 1601 patients, 617 of whom had at least one current diagnosis of anxiety or depression, 471 had a history of anxiety or depression, and 513 were controls with no history of anxiety or depression.

### Assessment

#### Composite Interview Diagnostic Instrument (CIDI)

The CIDI (version 2.1) is an interview that classifies psychiatric diagnoses according to the DSM-IV [23]. It is a widely used interview, which has good interrater reliability [24], high test-retest reliability [25], and high validity for the classification of depressive and anxiety disorders [26,27]. CIDI interviews were conducted by specifically trained research assistants. The CIDI classifies diagnoses that were present at some point in the patients' life (lifetime diagnoses), in the past half year and in the past month.

#### Beck Anxiety Inventory (BAI)

The BAI is a short list describing 21 anxiety symptoms such as "wobbliness in legs", "scared" and "fear of losing control" [8]. Respondents are asked to rate how much each of these symptoms bothered them in the past week, on a scale ranging from 0 (not at all) tot 3 (severely, I could barely stand it). The total score has a minimum of 0 and a maximum of 63. The scale was validated in a sample of 160 psychiatric outpatients with various anxiety and depressive disorders, diagnosed with the Structured Clinical Interview for DSM-III [28]. The BAI has a high internal consistency (Cronbachs *α *= .92) and a test-retest reliability over one week of .75 [8].

### Statistical analysis

All analyses were conducted in SPSS version 15.0 [29]. Regression analysis was performed to examine differences between group scores. The analyses were corrected for age and gender, because age was differentially distributed over the diagnostic groups and because female patients scored significantly higher than male patients in the total sample. All variables were entered simultaneously into the analysis. The analyses were repeated with different groups as the reference group to be able to compare all groups.

## Results

### Descriptive statistics

The average age of the participants was 45.9 years and the majority of the patients were female (68.8%). Almost one third of the participants had been diagnosed with an anxiety disorder in the past month (*N *= 493, 30.8%). Table 1 shows the age, gender and DSM-IV diagnosis of the participants.

**Table 1:** Age, gender and current DSM-IV diagnoses of participants (N = 1601)

	N	%
**All participants**	1601	
Age [range]	45.8 [18-65]	
Female gender	1102	68.8%

**Any anxiety disorder**	**493**	

Age [range]	45.7 [18-65]	
Female gender	346	70.2%
Social phobia*	68	*13.8%*
Panic disorder with agoraphobia*	42	*8.5%*
Panic without agoraphobia*	28	*5.7%*
Agoraphobia*	42	*8.5%*
Generalized anxiety disorder*	34	*6.9%*
>1 anxiety disorder	76	*15.4%*
Co-morbid anxiety & depression	203	*41.2%*

**Any depressive disorder**	**327**	

Age [Range]	46.2 [18-64]	
Female gender	223	68.2%
Dysthymia*	8	*2.4%*
Major depression*	101	*30.9%*
>1 depressive disorder	15	*4.6%*
Co-morbid depression & anxiety	203	*62.1%*

**Patients with a history of anxiety or depression**	471	*29.4%*

**Controls (no history of anxiety or depression)**	513	*32.0%*

*Disorder with no co-morbid anxiety disorder or co-morbid depressive disorder

Many patients with a diagnosis of an anxiety disorder had at least one co-morbid anxiety disorder. The percentage of patients with a co-morbid anxiety disorder varied over the diagnostic groups: anxiety co-morbidity was highest in patients with panic disorder or generalized anxiety disorder (54%) followed by patients with social phobia (51%) and patients with agoraphobia alone (35%). Almost half (41%) of the patients with an anxiety disorder also suffered from a depressive disorder, while 62% of the patients with a depressive disorder were also diagnosed with an anxiety disorder.

### Anxiety disorders

Table 2 shows the mean BAI scores of the control group (no history of anxiety or depression), patients with one anxiety disorder and patients with multiple anxiety disorders. Patients with a co-morbid depression were excluded from this analysis (*n *= 203).

**Table 2:** Mean BAI scores of patients with different anxiety disorders (with no co-morbid depression) and controls

			
Diagnosis (past month)	N	M	SD
**Controls**	513	4.09	5.06
Social phobia*	68	12.97	9.03
Panic disorder with agoraphobia*	42	16.00	11.02
Panic disorder without agoraphobia*	28	13.04	6.61
Agoraphobia*	42	11.62	8.51
Generalized anxiety disorder*	34	13.15	5.67
**Multiple anxiety disorders**	76	18.54	8.54

*Single anxiety disorder diagnosis

Patients with an anxiety disorder scored significantly higher than the controls (*p *< 0.001) and patients with multiple anxiety disorders scored considerably higher than all other groups (*p *< .05). The mean BAI score of patients with a panic disorder and agoraphobia was significantly higher than the mean score of patients with social phobia (*p *= 0.03) or agoraphobia alone (*p *< 0.001).

### Anxiety and depressive disorders

Table 3 shows that the score of depressed patients approximates the score of anxious patients (*p *= .41). Patients with co-morbid anxiety-depression scored significantly higher than patients with either an anxiety disorder or a depressive disorder alone (*p *< 0.001).

**Table 3:** Mean BAI scores of patients with a depressive disorder, an anxiety disorder and co-morbid anxiety-depression

			
Diagnosis (past month)	N	M	SD
**Depressive disorder**	109	13.34	8.72
**Anxiety disorder**	214	13.94	8.69
**Co-morbid anxiety-depression**	203	21.89	10.95

## Discussion

The results of our study show that primary care patients with different anxiety disorders score significantly higher than patients with no anxiety or depressive disorder. These results suggest that the BAI does reflect general anxiety in primary care patients. With regard to the different diagnostic groups of anxiety disorders, we did partly confirm the strong focus of the BAI on panic symptoms [10,11]. Patients with a panic disorder and agoraphobia scored significantly higher than patients with agoraphobia alone or social phobia. However, patients with a panic disorder without agoraphobia did not score significantly higher than the other groups. The high scores of patients with a panic disorder and agoraphobia might thus be explained by the severity of this specific disorder. In other studies in which the BAI was used, greater differences were found between the group of patients with a panic disorder and other diagnostic groups [11-13,30,31]. One reason for this discrepancy in findings might be the setting in which studies took place. Most of the previous studies were conducted in treatment centres for anxiety disorders, while the participants in the present study were actively recruited in primary care, also including patients with previously undiagnosed anxiety or depression. It is likely that more primary care patients present with less severe forms of panic disorder. Indeed, the mean score of patients with panic disorder in the present study seems to be substantially lower than the scores reported in studies with secondary care patients [11,13,30,31] coming closer to the scores of patients with a panic disorder in an epidemiological sample [32]. Furthermore, in the analysis of the present study, patient groups were specifically selected on the basis of (the absence of) co-morbidity, thus resulting in pure diagnostic groups. This may have provided a more accurate estimate of the mean scores of specific patient groups.

Beck and colleagues [8] claimed that the BAI measures anxiety while minimizing its overlap with depression but this was not sustained by the results of the present study. For practical purposes, this is a two-sided finding. The BAI appears to be robust for depression, but not entirely specific for anxiety in a primary care population. These findings are consistent with the results of earlier studies that compared the total BAI scores of depressed and anxious patients [15,19]. Steer and colleagues relate their findings to the low co-morbidity rate in their sample, but this argument does not hold up in the present study. There could be several explanations why depressed patients score almost as high as anxiety patients. First of all, sub-threshold anxiety experienced by patients with a depressive disorder may have increased their anxiety scores. Sub-threshold anxiety was not assessed in the present study, but previous research has shown that a substantial number of depressed patients also experience some form of (sub-threshold) anxiety [33,34]. Secondly, somatoform disorders were not classified with the CIDI interview, while these disorders are prevalent in primary care patients with a depressive disorder, and can also cause the physiological symptoms described in the BAI [35]. A third explanation might be that anxiety and depression share a common underlying factor, often referred to as 'negative affect' [34,36]. There is longstanding debate about this question, growing stronger due to the pressure of the upcoming publication of the DSM-V and fuelled by the considerable prevalence of co-morbidity between anxiety and depression and the symptom overlap on anxiety and depression scales. With regard to this third hypothesis, the sensitivity of the BAI for shared symptomatology would be more of a quality than a deficiency. Fourthly, total scores for self-report questionnaires, in general, might not be precise enough to measure difficult constructs such as anxiety and depression. There is some evidence that the BAI is able to discriminate between anxiety and depression when items are weighted, as happens in factor analysis [19]. However, weighting the items would complicate the use of the BAI to such an extent that its use would not be feasible in primary care.

A strength of this study is the large size of this primary care sample, diagnosed with a valid interview identifying five different anxiety disorders and two depressive disorders. Because of the high prevalence of co-morbidity in patients with anxiety and depressive disorders, such a large sample is needed to compare (sub-)groups of patients with a specific anxiety or depressive disorder. However, even in this large sample, patients with one specific anxiety disorder are scarce, limiting the power of the analyses. Another limitation of the analysis was the skewed distribution of the scores. Although we considered performing a log transformation, we decided to use raw scores to facilitate the interpretability of the scores in clinical practice.

## Conclusions

The results indicate that the BAI reflects the severity of anxiety in primary care patients with different anxiety disorders. The use of questionnaires such as the BAI may improve the care that is provided and is desirable from the viewpoint of primary care patients [5]. However, as the use of questionnaires in primary care is not common practice, this should be stimulated by means of guidelines, training and education. Further research will be needed to evaluate the usefulness of the BAI in monitoring the severity of anxiety during treatment and over time. In addition, researchers should establish criteria for improvement and remission according to the BAI score, in primary care patients. When questionnaires such as the BAI are used within a framework of care, such as case management or collaborative care, they will optimally help to improve the treatment of primary care patients with anxiety disorders [3,37,38].

## Competing interests

The authors declare that they have no competing interests.

## Authors' contributions

AM participated in the design of the study, performed the statistical analyses and drafted the manuscript. CFC, HvM, PS and AvB participated in the design of the study, helped drafting the manuscript and critically commented on the manuscript. BP obtained funding for and designed the NESDA study, supervised data collection and critically commented on the manuscript. All authors read and approved the final manuscript.

## Authors' information

AM is a PhD student working on a dissertation about collaborative care for anxiety disorders in primary care. CFC is full professor of Social Psychiatry and Principal Investigator of several randomized clinical trials on collaborative care. HvM is associate professor of general practice, he participated in several national guideline committees on mental health, and is a practising GP. PS is professor in Clinical Psychology and was chairman of the Dutch Committee Multidisciplinary Guidelines for Anxiety Disorders in Mental Health Care. BP is Principal Investigator of the NESDA study, and professor of Psychiatric Epidemiology. AvB is professor Evidence-based Psychiatry. He performed meta-analyses, systematic (Cochrane) reviews and randomized controlled trials (RCT's) in patients with anxiety disorders.

## Pre-publication history

The pre-publication history for this paper can be accessed here:

http://www.biomedcentral.com/1471-2296/12/66/prepub
